# Ascaris Peritonitis in a Pediatric Patient With Typhoid Fever

**DOI:** 10.7759/cureus.81813

**Published:** 2025-04-06

**Authors:** Charles Tumwesige, Arthur J Nek, Daniel Odulusi, Colin Meghoo, Johnson Ebine

**Affiliations:** 1 General Surgery, Kabale University School of Medicine, Kabale, UGA; 2 Surgery, CHI Good Samaritan Hospital, Kearney, USA; 3 General Surgery, Kabale Regional Referral Hospital, Kabale, UGA

**Keywords:** ascariasis, intestinal perforation, laparotomy, peritonitis, typhoid fever

## Abstract

Intestinal infestation with ascaris worms is very common worldwide and usually causes minimal or no abdominal symptoms. Severe abdominal complications such as bowel obstruction may occur, but bowel perforation from ascariasis is rare. We present a case of a seven-year-old boy who initially presented with typhoid fever and then developed ascaris peritonitis requiring emergent surgery. We discuss preoperative diagnosis and perioperative management, as well as the purported mechanisms of coinfection with typhoid fever and ascariasis that can lead to small bowel perforation.

## Introduction

Ascariasis is the most common parasitic infestation worldwide, affecting up to 1.5 billion people [1]. The disease is caused by the nematode *Ascaris lumbricoides *(commonly known as roundworm) and primarily affects preschool-aged children from impoverished backgrounds [2].

Southwestern Uganda, where the patient in this case report stays, has an even higher burden of soil-transmitted helminths. The prevalence of helminthic infestation among preschool, school-aged children, and women of reproductive age in one of the districts in this region (Kisoro district) was found to range from 49.4 to 60.6%, with *Ascaris lumbricoides* and *Trichuris trichiura *being most prevalent [3].

Patients infested with Ascarid worms are usually asymptomatic, but may develop varied symptoms such as weight loss, growth impairment, malnutrition, nausea, vomiting, and diarrhea [4]. Transoral or transanal passage of live worms in stool can occur, and rare occurrences of biliary obstruction, pancreatitis, intestinal obstruction, and perforation can develop from errant migration of adult worms [5].

The most frequent surgical presentation of ascariasis is with intestinal obstruction, often resulting from a large burden of worms occluding the intestinal lumen [5]. Bowel perforations due to ascaris infestation are rarely reported and seem more likely to occur in conjunction with other intestinal conditions such as amoebiasis, regional enteritis, typhoid fever, tuberculosis, lymphoma, or recent abdominal trauma [1, 2, 6].

Concurrent infection with typhoid fever and a helminthic infestation can be seen in Sub-Saharan Africa and other regions with low socioeconomic conditions where fecal oral transmission of disease is more likely [7]. Typhoid fever is a bacterial infection caused by gram-negative bacteria, either* Salmonella *Typhi or *Salmonella* Paratyphi, that commonly causes inflammation of the small intestines, and in particular the distal ileum, and can itself cause small bowel perforation.

We present a rare case of ileal perforation secondary to ascariasis infestation and typhoid co-infection in a seven-year-old child in Kabale district, southwestern Uganda. This case illustrates the potential for a synergistic interaction between typhoid-induced intestinal wall inflammation and the mechanical effects of ascariasis, resulting in a life-threatening intestinal perforation.

## Case presentation

A seven-year-old male initially presented to a local health center in southwestern Uganda with a three-day history of abdominal pain, distension, and vomiting, associated with low-grade fevers and dizziness. He was diagnosed with typhoid fever by Widal test (1:240 dilution), and he was started on intravenous antibiotics, pain medications, and antipyretics. By the second day of treatment, he developed worsening abdominal symptoms, and an abdominal ultrasound was obtained. Findings on the sonogram revealed free fluid in the peritoneum with extra luminal mobile worms suggestive of gut perforation. 

The patient was sent to the regional referral hospital in Kabale, where he was noted to be ill-appearing, afebrile, with an elevated pulse (124bpm) and a normal blood pressure, and mild tachypnea without respiratory distress. He had mild dehydration, pink conjunctiva, white sclera, and no appreciable rose spots. He had evidence of mild chronic malnutrition for age (Z score between -1 and -2). An abdominal exam revealed moderate abdominal distension, with reduced bowel sounds, diffuse tenderness, and abdominal guarding. The remainder of the systemic examination was normal.

Complete blood count (Table 1) revealed leukopenia, lymphocytopenia, normal eosinophil count, and normal hemoglobin level. Liver and kidney function tests were normal except for hypoalbuminemia (Table 2). A typhoid IgM test was later performed, with positive results supporting the Widal test. Salmonella cultures were unavailable.

**Table 1:** Complete blood count

Parameter	Result	Reference range
WBC	2.33 x 10^3^/ul	3-15 x 10^3^/ul
Neutrophils	1.66 x 10^3^/ul	1.5-7.0 x10^3^/ul
Lymphocytes	0.55 x 10^3^/ul	1-3.7 x 10^9^/ul
Eosinophils	0.00 x 10^3^ul	0.00-0.40 x 10^3^ul
Hemoglobin	10.9g/dl	8.0-17.0g/dl
Platelets	101 x10^3^/ul	150-450ul

**Table 2:** Renal and liver function tests

Parameter	Result	Reference range	Unit
Urea	39.9	10-50	mg dL
Creatinine	0.83	0.6-1.1	mg/dL
Albumin	2.01	3.8-5.1	g/dL
Amylase	68	0-220	U/L
Bilirubin direct	0.18	0-0.2	mg dL
Glucose	81.8	75-112	mg dL
ASAT GOT	14	0-37	U/L
ALAT / GPT	41	0-42	U/L
Bilirubin total	0.52	0.1-1.2	mg / dL
Total protein	6.6	6.6-8.7	g/dL

A clinical diagnosis of peritonitis was made, and pre-operative resuscitation with isotonic fluids and pre-operative antibiotics was administered, as per age and body weight dosing recommendations. The patient was taken urgently to the operation theater for an exploratory laparotomy.

**Figure 1:**
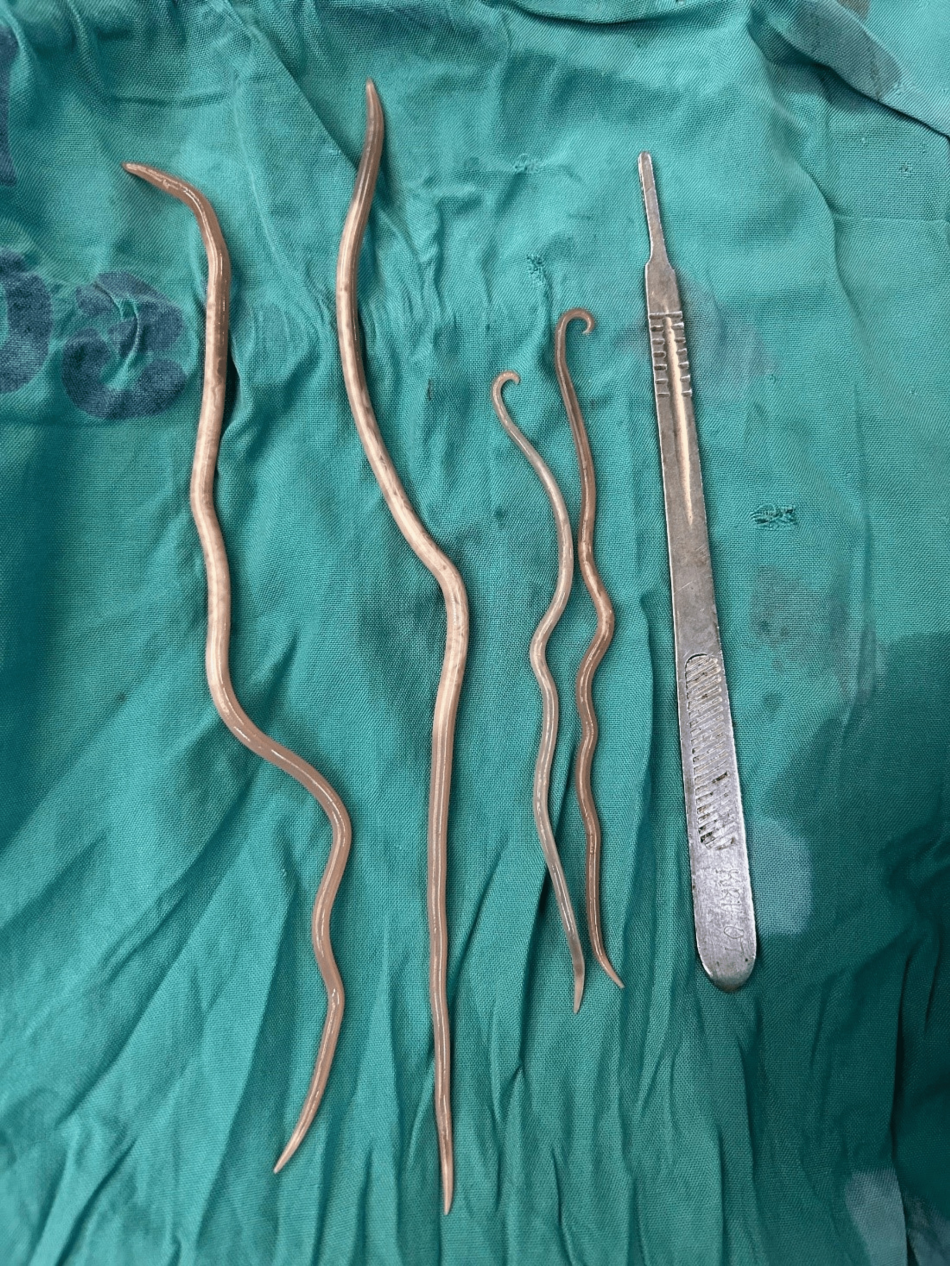
Four ascaris worms removed from peritoneum, with long knife handle for scale

**Figure 2:**
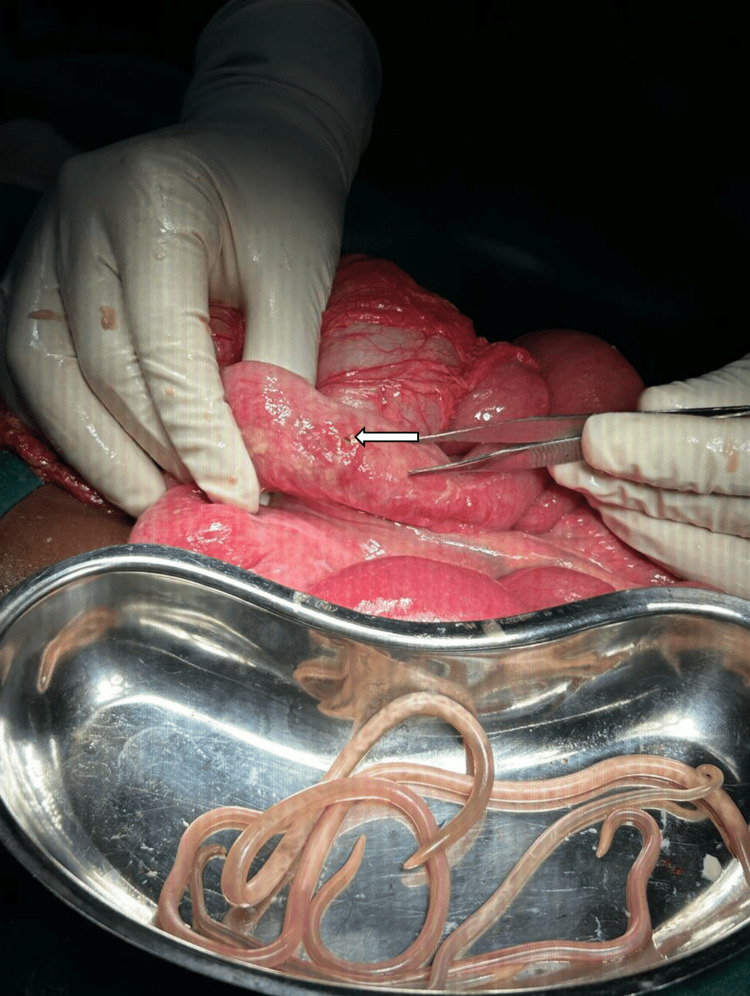
Area of small bowel perforation in the distal ileum (arrow) with worms extracted from the peritoneum in the foreground

Intraoperatively, approximately 1000 ml of non-purulent serous ascites containing four live adult worms were noted in the peritoneal cavity and removed (Figure 1). There was mild distal small bowel serositis with associated mesenteric lymphadenopathy. There was no bowel distension or ischemia. An antimesenteric small bowel perforation of 4 mm diameter was found 35 cm from the ileocecal junction with surrounding fibrinous debris (Figure 2). No intraluminal or intramural mass was noted at the site of the perforation. A few scattered worms were palpable within the lumen of the proximal small bowel and were not removed. Sharp debridement of the edges of the perforation was performed, and the bowel was repaired primarily in two layers using Vicryl 3.0 with the continuous suturing technique. The peritoneum was lavaged with warm normal saline, and the abdomen was closed without drains.

Postoperative antibiotics (which aimed to target typhoid infection as well) were administered along with an oral antihelminthic (mebendazole 500mg once daily for three consecutive days). One dose of hydrocortisone was given to reduce the possible risk of anaphylaxis from free *Ascaris lumbricoides* worms. Family members were also given antihelminthic medication in addition to education about good hygiene and sanitation. The patient’s postoperative course was complicated by a superficial surgical site infection that was managed with local wound care and nutritional supplementation. The patient was discharged home on postoperative Day 21 with a healed midline wound.

## Discussion

The diagnosis of bowel perforation, regardless of its cause, begins with a thorough history and physical examination, which can be supported by imaging studies. A plain abdominal X-ray may reveal an intraluminal worm bolus, findings of intestinal obstruction, or pneumoperitoneum. Occasionally, these radiographs can be non-diagnostic, and advanced imaging or careful clinical consideration is warranted [2].

Abdominal ultrasound can be diagnostic in an acute abdomen secondary to ascariasis [8]. It can identify worms in the lumen of the bowel, in the biliary tree, or free in the peritoneum in the case of intestinal perforation and ascaris peritonitis [9, 10]. Sonographically, these worms appear as curvilinear or serpentine structures without posterior acoustic enhancement, and their gut typically manifests the “triple line sign” as two parallel echogenic lines with a central anechoic region [11].

Bowel perforation as a complication of ascaris infestation is uncommon [1, 12] with few cases published. From Efem’s review [12], intestinal perforation from ascaris can be categorized into two etiologic types: primary and secondary. In primary perforation, the worm directly penetrates an otherwise healthy segment of intestine, while in the more common secondary perforation, an underlying condition first weakens a section of intestine, rendering it susceptible to perforation by ascaris worms. Such coexisting conditions can include amoebiasis, typhoid fever, Meckel’s diverticulitis, ischemic necrosis from volvulus, lymphomas, abdominal traumatic injuries, or inflammatory bowel diseases [6, 12, 8].

Rarely, ascarid peritonitis has been reported arising from perforations of the gallbladder, stomach, appendix, or a Meckel’s diverticulum. [4, 5, 8, 9, 13]. Small bowel perforations are more common and usually occur in the distal ileum, despite the typical burden of ascaris intraluminal infection in the jejunum [12, 14]. Typhoid infection of the bowel is known to affect the distal 60 centimeters of the ileum, where a high concentration of vulnerable Peyer’s patches are located, and this accounts for the frequency of typhoid perforation in this location [14]. As with our patient with dual infection of typhoid fever and ascariasis, this also explains the common location of the ascaris bowel perforation at the site of the terminal ileum weakened by typhoid fever.

Urgent surgical intervention is indicated in patients with ascariasis peritonitis in order to control the peritonitis or to identify and repair or resect the site of perforation [1, 15]. The two surgical techniques commonly used to manage enteric perforations are primary repair and intestinal resection with anastomosis [14]. Primary repair may be preferable in cases of one or a few widely spaced small perforations without associated bowel necrosis or ischemia, but resection with anastomosis can be considered if the perforations are many and close together or involving a severely diseased segment of bowel [14] as can be seen in many cases of typhoid perforation. Ileostomy may be considered in cases of gross peritoneal contamination or severe sepsis.

Damaging the live ascaris worms in the peritoneum at the time of operation can cause an anaphylactic reaction, so care should be taken to remove the adult worms intact [16]. The presence of dead worms in the peritoneum or their eggs can trigger the body’s immune reaction, leading to granulomatous peritonitis [13, 17]. This supports a careful search for and removal of all intraperitoneal worms at the time of operative intervention.

## Conclusions

While most intestinal infestations with adult ascaris worms are asymptomatic, life-threatening complications requiring emergency surgical intervention can occur. In evaluating patients with an acute abdomen and preoperative or intraoperative findings of ascaris peritonitis, concomitant bowel damage from infection, trauma, or neoplasm should be considered. 

A high index of suspicion of coexisting typhoid fever infection should be maintained, especially in children living in areas where ascariasis and typhoid fever are endemic. The interplay between typhoid-induced intestinal wall compromise and the mechanical effects of ascariasis can result in small bowel rupture in the distal ileum. Routine treatment of children for helminthiasis as well as immunization, early treatment of typhoid fever, and good sanitation and personal hygiene practices can prevent typhoid and ascarid diseases and their potentially catastrophic surgical complications.
